# Hydrophilic Chlorin e6-Poly(amidoamine) Dendrimer Nanoconjugates for Enhanced Photodynamic Therapy

**DOI:** 10.3390/nano8060445

**Published:** 2018-06-18

**Authors:** So-Ri Lee, Young-Jin Kim

**Affiliations:** Department of Biomedical Engineering, Daegu Catholic University, Gyeongsan 38430, Korea; esdkiss@naver.com

**Keywords:** photodynamic therapy, chlorin e6, poly(amidoamine) dendrimer, nanoconjugate, cervical cancer

## Abstract

In photodynamic therapy (PDT), chlorin e6 (Ce6), with its high phototoxic potential and strong absorption of visible light, penetrates deeply into photodamaged tissue. However, despite this fact, the direct application of Ce6 to PDT has been limited by its low water solubility and poor cancer cell localization. To ameliorate this situation, we report herein on the use of a hydrophilic nanoconjugate (DC) comprised of Ce6 and poly(amidoamine) dendrimer, which improves the water solubility and intracellular uptake of Ce6, thereby enhancing PDT efficacy. The synthesis of DC was verified by ^1^H nuclear magnetic resonance (NMR) analysis, and the coupling ratio of Ce6 introduced onto DC was 2.64. The prepared DC was spherical, with an average diameter of 61.7 ± 3.5 nm. In addition, the characteristic ultraviolet-visible absorption bands of DC in distilled water were similar to those of free Ce6 in dimethyl sulfoxide (DMSO), indicating that the Ce6 chromophore did not change upon conjugation. Investigation using fluorescence spectroscopy and confocal microscopy revealed a greater intracellular uptake of DC than of Ce6 alone. Moreover, DC exhibited significantly increased phototoxicity to human cervical cancer cells, mostly because of apoptotic cell death. These results imply that DC is a candidate for the clinical treatment of PDT.

## 1. Introduction

Photodynamic therapy (PDT) has emerged as an innovative therapeutic method for treating various oncological diseases, including gastrointestinal, esophagus, bladder and cervical cancer [1,2,3]. This therapeutic modality is considered superior to traditional therapies such as surgery, radiotherapy and chemotherapy because it involves a minimally-invasive clinical procedure. In addition, traditional chemotherapy usually has serious side effects, and its clinical application is confined by multidrug resistance [2]. However, PDT is expected to overcome multidrug resistance because the PDT cytotoxicity mechanism for cancer cells differs from that of traditional chemotherapy.

PDT is based on the accumulation of a photosensitizer in specific target cells, followed by selective optical irradiation of an appropriate wavelength to generate a highly reactive oxygen species (ROS), such as singlet oxygen. Increasing the ROS above a critical concentration threshold allows it to react directly with the biological substrate, oxidizing vital cellular components and inducing an acute cell-stress response, culminating in cellular death, mainly by apoptosis and/or necrosis [4]. Therefore, the efficacy of PDT depends primarily on the intracellular accumulation of the photosensitizer in the target cells, in which the intracellular accumulation of the photosensitizer is affected by its chemical properties.

Unfortunately, such photosensitizers are limited in clinical use because of prolonged photosensitivity, nonspecific phototoxicity for normal cells due to insufficient selective accumulation around the specific target cells and hydrophobicity [5]. In particular, hydrophobic photosensitizers can easily precipitate out or adhere to normal cell surfaces in the biological environment, losing their PDT efficacy and causing severe side effects. To address this situation, various photosensitizer-delivery systems have been investigated, such as polymeric micelles and conjugates, which have provided highly soluble photosensitizers in aqueous solutions and have improved tumor specificity [6,7]. One such system, photosensitizer-loaded cationic polymer complexes, combines with negatively-charged cell membranes, resulting in accelerated cellular internalization of photosensitizers [5,8].

Chlorins are a class of plant-derived tetrapyrroles and are prospective photosensitizers because of their high phototoxic potential and remarkably strong absorption in the red [9]. These characteristics of chlorins allow for deeper tissue photodamage, which is particularly essential for treating cancer. However, in general, chlorins exhibit high hydrophobicity and low solubility in aqueous solutions, causing them to accumulate in the skin. In addition, they have prolonged light sensitivity, on the order of weeks, so that they continue to be active long after PDT treatment. Poly(amidoamine) (PAMAM) dendrimers are nanoscale monodisperse macromolecules with a hyperbranched three-dimensional architecture and a large number of terminal function groups [5,10]. PAMAM dendrimers have been used in various drug-delivery systems because they not only spatially distribute the drug, but also maintain superior dispersion stability even under physiological conditions [10].

**Figure 1:**
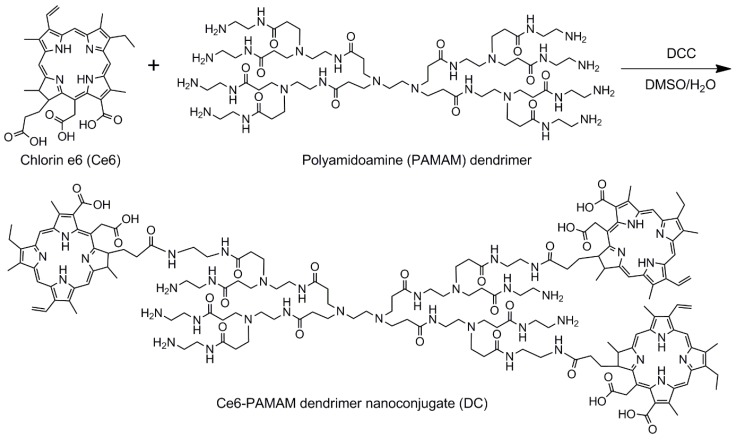
Schematic diagram of synthesis of hydrophilic Ce6-PAMAM dendrimer nanoconjugate (DC).

Cervical cancer is the third most common cancer among women worldwide, and the causal factor of cervical cancer is infection by the human papilloma virus [11]. This persistent viral infection leads to intraepithelial transformations of an insidious and progressive nature that culminates in a cancer if left untreated. PDT may be an alternative to the traditional invasive treatment of cervical cancer [12,13,14]. Therefore, the development of hydrophilic chlorin conjugates may lead to a successful treatment for cervical cancer. Motivated by this goal, the main purpose of the present study is to enhance PDT efficacy of chlorin e6 (Ce6) by enhancing its water solubility and cellular internalization. Toward this end, we prepared a hydrophilic Ce6-PAMAM dendrimer nanoconjugate (DC) (Figure 1). The morphology, chemical structure and hydrophilicity of the prepared DC were systematically examined, and the cellular internalization and phototoxicity of DC were evaluated on the human cervical cancer cell line (HeLa) by using fluorescence microscopy and MTT assays.

## 2. Materials and Methods

### 2.1. Materials

Ce6 was purchased from Frontier Scientific (Logan, UT, USA). Poly(amidoamine) (PAMAM) dendrimer (ethylenediamine core, Generation 1.0), *N*,*N*’-dicyclohexylcarbodiimide (DCC), 3-(4,5-dimethyl-2-thiazolyl)-2,5-diphenyl-2H-tetrazolium bromide (MTT), 3-amino-7-dimethylamino-2-methylphenazine hydrochloride (neutral red), bisbenzimide H 33258 (Hoechst 33258, H-33258), 9,10-dimethylnathracene (DMA), *N*,*N*-dimethylformamide (DMF) and dimethyl sulfoxide (DMSO) were obtained from Sigma-Aldrich (St. Louis, MO, USA) and used without further purification. The human cervical cancer cell line (HeLa) was obtained from the Korean Cell Line Bank (KCLB, Seoul, Korea). Dulbecco’s modified Eagle’s medium (DMEM), fetal bovine serum (FBS), penicillin-streptomycin and Dulbecco’s phosphate-buffered saline (DPBS, pH 7.4) were obtained from Gibco BRL (Waltham, MA, USA). SlowFade Gold antifade mountant, LIVE/DEAD Viability/Cytotoxicity Assay Kit and the Image-iT LIVE Green Reactive Oxygen Species Detection Kit were purchased from Molecular Probes (Eugene, OR, USA). The Actin Cytoskeleton and Focal Adhesion Staining Kit was purchased from Merck Millipore (Burlington, MA, USA). Other reagents and solvents were commercially available and used as received.

### 2.2. Synthesis of Ce6-PAMAM Dendrimer Nanoconjugate

The hydrophilic Ce6-PAMAM dendrimer nanoconjugate (DC) was synthesized as follows. First, 98.5 mg Ce6 were dissolved in 20 mL DMSO, followed by the addition of 51.1 mg DCC to activate the carboxylic groups. After adding to the mixture 357.5 mg PAMAM dendrimer in 30 mL DMSO/distilled water (DW) at a ratio of 7:3, the reaction was allowed to progress at room temperature for 24 h to produce DC. The products were filtered and isolated by using a tubular membrane dialysis in a mixture of DMSO and DW for 48 h to remove unreacted agents, followed by lipophilization in vacuo. To determine the amount of Ce6 conjugated to DC, the absorbance was measured at 404 nm by using an ultraviolet-visible (UV-visible) spectrometer according to the previous studies [7,15]. Free Ce6 dissolved in DMSO was prepared at different concentrations (1, 2, 4, 8, 12 and 20 μg/mL) to generate a standard curve, and the amount of Ce6 conjugated to PAMAM dendrimer was measured as the absorbance at 404 nm by using a UV-visible spectrometer.

^1^H NMR (400 MHz, DMSO-d_6_), δ (ppm) = 9.47, 9.69, 9.11 (m, H-meso), 8.33 (d, CH=CH_2_), 6.39, 6.16 (d, CH=CH_2_), 5.59 (d, chlorin-CH_2_CO), 4.56, 4.47 (s, CCH), 3.83 (m, CH_2_CH_3_), 3.53, 3.33 (s, chlorin-CH_3_), 2.58 (m, CH_2_CH_2_CO), 2.32 (m, chlorin-CH_2_CH_2_CO), 1.60 (t, chlorin-CH_2_CH_3_) for Ce6, 3.03 (m, NHCH_2_CH_2_N) 2.84 (m, NCH_2_CH_2_CO), 2.63 (m, NHCH_2_CH_2_N), 2.18 (NCH_2_CH_2_CO) for the PAMAM dendrimer.

### 2.3. Characterization of Ce6-PAMAM Dendrimer Nanoconjugate

The structure of DC and its degree of Ce6 conjugation were determined by ^1^H nuclear magnetic resonance (NMR) spectroscopy (ADVANCE III 400, Bruker, Billerica, MA, USA), and its morphology was determined by transmission electron microscopy (TEM, H-7600, Hitachi, Tokyo, Japan). The average diameter of nanoconjugates was determined by analyzing the TEM images with Image-Pro Plus (Media Cybernetics Inc., Rockville, MD, USA). The particle size distribution was also measured by the dynamic light scattering (DLS) technique using a Zetasizer Nano ZS (Malvern Instruments, Malvern, UK). UV-visible spectra were recorded on a Hitachi U-2900 spectrometer (Tokyo, Japan), and the fluorescence emission spectra were measured with a PerkinElmer LS55 spectrofluorophotometer (Waltham, MA, USA) at 25 °C.

### 2.4. Singlet Oxygen Detection

The singlet oxygen (^1^O_2_) generation was measured by an indirect method using a chemical probe [16]. In this study, ^1^O_2_ generation from free Ce6 or DC was detected using DMA as the ^1^O_2_ probe. Free Ce6 or DC (1 mg/mL Ce6) was first dissolved in DMSO to prepare stock solution. This stock solution was dispersed in DPBS (pH 7.4) to obtain a concentration of 4 μg/mL Ce6, and then, DMA stock solution (20 mM in DMF) was added to give a final concentration of 20 μM DMA. Samples containing drug and DMA were irradiated at a light intensity with a 2.5-J/cm^2^, 671-nm laser beam (LVI Technologies, Anyang, Korea). The fluorescence spectra of DMA (excitation, 360 nm; emission, 380–540 nm) as a result of the photosensitization reaction were monitored with a Perkin-Elmer LS55 spectrofluorophotometer (Waltham, MA, USA). The change in DMA fluorescence intensity (F_f_ − F_s_) was plotted after subtracting each sample fluorescence intensity (F_s_) from the full DMA fluorescence intensity (without free Ce6 or DC, indicating no singlet oxygen, F_f_).

### 2.5. Cell Culture and Incubation Conditions

All experiments used the human cervical cancer cell line (HeLa). HeLa cells were cultured in DMEM containing 10% FBS and 0.5% penicillin-streptomycin and were incubated at 37 °C in a humidified 5% CO_2_ atmosphere. When the cells reached 80% confluence, they were harvested by using 0.25% trypsin-ethylenediaminetetraacetic acid (EDTA) and seeded in a new tissue culture plate to produce a subculture. DC and free Ce6 were dispersed in a serum-free medium. Untreated cells or cells that were maintained in the dark were used as reference cells.

### 2.6. Intracellular Uptake and Distribution Tests

A multimode microplate reader (VICTOR3, PerkinElmer, Waltham, MA, USA) equipped with n excitation filter of 405 nm and an emission filter of 665 nm was used to quantify the intracellular uptake of free Ce6 and DC. HeLa cells (1 × 10^4^ cells/well) were seeded into 96-well plates in 1 mL of culture medium containing 10% FBS and were incubated for 24 h. These cells were treated with free Ce6 or DC (4 μg/mL Ce6) for 1–4 h, following which the cells were washed twice with DPBS. After optically exciting the cell samples, the fluorescence emission of Ce6 or DC was measured by using the multimode microplate reader.

The intracellular uptake of drugs in cancer cells was determined by using confocal laser scanning microscopy. HeLa cells (1 × 10^4^ cells/well) were seeded into an 8-well chamber slide for 24 h before being treated with the drug. The cells were incubated with free Ce6 or DC (4 μg/mL Ce6) for 2 h and rinsed twice with DPBS. Next, the cells were fixed in 4% paraformaldehyde solution for 10 min and permeabilized by applying 1% triton X-100 for 3 min. The cells were treated with 10 μg/mL H-33258 to stain the cell nuclei and, to label actin, with 3 μg/mL phalloidin-tetramethylrhodamine (TRITC) for 30 min at room temperature in the dark. The intracellular uptake and distribution of drugs were determined by using an inverted LSM 700 confocal laser scanning microscope (Carl Zeiss, Oberkochen, Germany).

### 2.7. Cell Phototoxicity Assay

To determine cell viability in the dark, HeLa cells (1 × 10^4^ cells/well) were seeded into 96-well plates and incubated for 24 h at 37 °C. After cell stabilization, the culture medium was replaced with 200 μL of culture medium containing free Ce6 or DC (0–4 μg/mL Ce6), followed by incubation for 2 h. The cells were then washed twice with a serum-free medium, and cell viability was evaluated via an MTT assay after 24 h.

To determine in vitro phototoxicity after laser irradiation, HeLa cells (1 × 10^4^ cells/well) were seeded into 96-well plates and incubated for 24 h at 37 °C. These cells were then treated with free Ce6 or DC (0–4 μg/mL Ce6). After incubation for 2 h, the cells were washed twice with a serum-free medium and irradiated with a 2.5-J/cm^2^, 671-nm laser beam. After incubation for 24 h, the viability of irradiated cells was evaluated via an MTT assay.

A qualitative cell viability assay was done by using the LIVE/DEAD Viability/Cytotoxicity Assay Kit. The kit contains calcein AM and ethidium homodimer-1 (EthD-1). Calcein AM stains live cells green, whereas EthD-1 stains dead cells red [17]. HeLa cells (5 × 10^4^ cells/well) were seeded into an8-well chamber slide and incubated for 24 h at 37 °C. Next, these cells were treated with free Ce6 or DC (4 μg/mL of Ce6). After incubation for 2 h, the cellular layers on the sample surface were rinsed twice with DPBS and irradiated with a 2.5-J/cm^2^, 671-nm diode laser. Afterwards, the cells were treated for 20 min at 37 °C with 1 μM of calcein AM and 2 μM of EthD-1 to determine cell viability after 24 h of incubation. Finally, the cells were observed by using an inverted fluorescence microscope (Eclipse TS100, FITC-G2A filters, Nikon, Tokyo, Japan) equipped with a cooled charge-coupled device (CCD) camera (DS-U2, Nikon, Japan) and with NIS-Elements Imaging Software.

### 2.8. Generation of Reactive Oxygen Species

The generation of the ROS was monitored by using the Image-iT LIVE Green Reactive Oxygen Species Detection Kit, which contains 5-(and-6)-carboxy-2′,7′-dichlorodihydrofluorescein diacetate (carboxy-H2DCFDA). H2DCFDA is a nonfluorescent penetrant that permeates live cells and is hydrolyzed by intracellular esterases to form H2DCF. In the presence of the ROS, H2DCF oxidizes in the cytoplasm and is converted to DCF, which can emit green fluorescence [2]. HeLa cells (5 × 10^4^ cells/well) were seeded into 24-well plates and incubated for 24 h at 37 °C, following which, they were treated with Ce6 or DC (4 μg/mL Ce6). After incubation for 2 h, the cells were rinsed twice with Hank’s balanced salt solution and irradiated with a 2.5-J/cm^2^, 671-nm diode laser. Next, the cells were treated with 5 μM carboxy-H2DCFDA for 30 min at 37 °C, and the concentration of intracellular ROS trapped by DCF was determined quantitatively by using a flow cytometer (FACSCalibur™, BD Biosciences, Franklin Lakes, NJ, USA). Data were analyzed by using Cellquest Software (BD Biosciences, Franklin Lakes, NJ, USA).

### 2.9. Apoptotic Analysis

Morphological changes after photodynamic treatment were assessed by visualizing the control and drug-treated cells by using fluorescence microscopy. HeLa cells (2 × 10^4^ cells/well) were seeded into an 8-well chamber slide for 24 h before being treated with the drug. The cells were incubated with free Ce6 or DC (4 μg/mL Ce6) for 2 h, rinsed twice with DPBS and illuminated with a 2.5-J/cm^2^, 671-nm diode laser. After 24 h, the cells were fixed in 4% paraformaldehyde solution for 10 min and stained with l mL of neutral red (30 μg/mL) or with 1 mL of H-33258 (10 μg/mL) for 30 min at room temperature in the dark. After washing and air drying, a coverslip was mounted on a microscope slide with a drop of antifade mounting solution to reduce fluorescence photobleaching. The cells were observed by using an inverted fluorescence microscope.

## 3. Results and Discussion

### 3.1. Synthesis and Characterization of DC Nanoconjugate

To prepare the hydrophilic photosensitizer, DC, Ce6 was conjugated to the water-soluble PAMAM dendrimer by a simple one-step reaction of the amino and carboxyl groups using DCC (Figure 1). The structure of DC was determined by ^1^H NMR spectroscopy, and the result showed the expected characteristic peaks of DC (Figure S1). The characteristic peaks at 9.47–9.11, 8.33, 6.39, 6.16, 5.59, 4.56, 4.47, 3.83, 3.53, 3.33, 2.58, 2.32 and 1.60 ppm were all associated with Ce6. In addition, the signals ascribed to the PAMAM dendrimer appeared at 3.03, 2.84, 2.63 and 2.18 ppm in the ^1^H NMR spectrum of DC. The coupling ratio of Ce6 that was introduced to DC was calculated by assignment of ^1^H NMR peaks. As a result, the Ce6 coupling ratio was 2.61, in which the Ce6 molar feed ratio was three. In addition, the coupling ratio of Ce6 was also calculated based on the UV-visible absorption spectrum, and it was 2.64.

**Figure 2:**
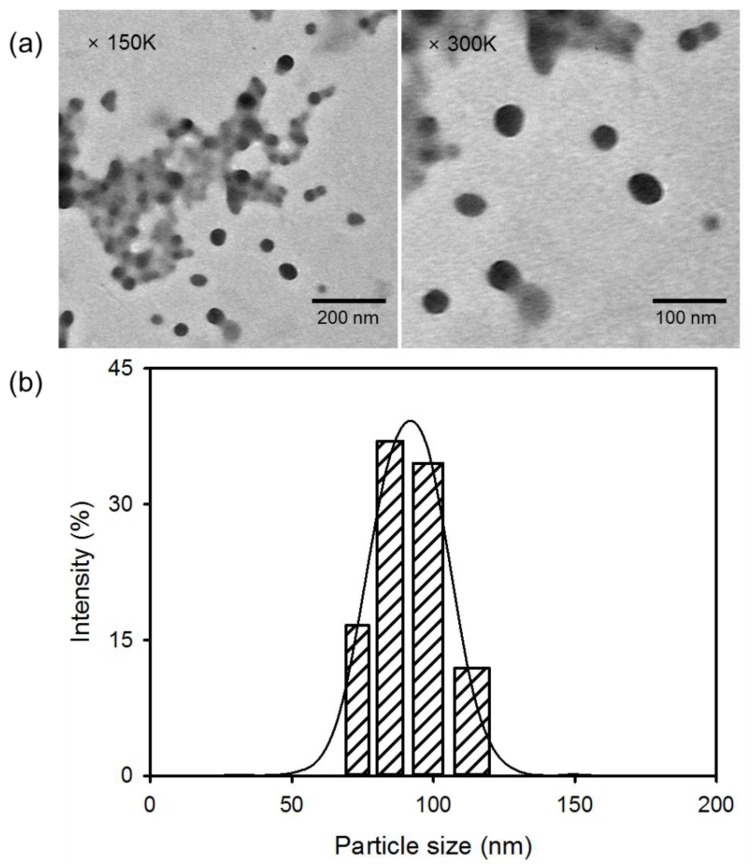
(**a**) TEM images and (**b**) particle size distribution of DC.

Figure 2a shows the morphological structure of DC measured by TEM. The TEM images reveal spherical DC with an average diameter of 61.7 ± 3.5 nm. DC self-aggregates to form nanosized conjugates through intermolecular interactions such as hydrogen bonding and ionic interactions. The PAMAM dendrimer contains a large number of terminal functional groups with a positive charge [5]. Positively-charged groups on DC molecules can provide binding sites, thereby leading to the accumulation of Ce6 moieties due to ionic interactions. In addition, specific stereochemical arrangements can form, and the charge distribution of reactive groups in complexes comprising PAMAM and Ce6 moieties can proceed. Moreover, intermolecular hydrogen bonding in DC also affected the formation of nanosized conjugates. The size distribution of DC, as determined by DLS, revealed a unimodal pattern with a diameter of 90.7 ± 8.9 nm; this was approximately 29 nm larger than the size observed by TEM (Figure 2b). The particle size measured by DLS was influenced by the dynamic diameter of the micelle structure, which was swollen in the aqueous solution. However, DC nanoconjugates that were determined by TEM were in the dried state. These results indicate that DC less than 100 nm in size may be considered suitable for the accumulation of Ce6 at the targeted tumor.

**Figure 3:**
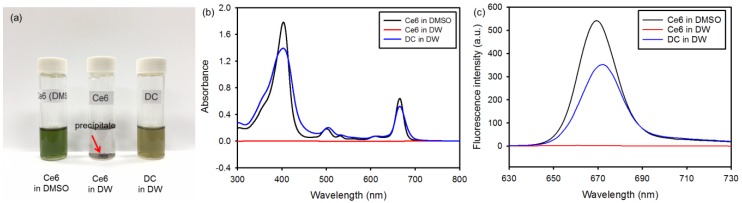
(**a**) Photographs of free chlorin e6 (Ce6) and DC dissolved in DMSO or DW (0.2 mg/mL Ce6). The arrow shows the precipitate of Ce6. (**b**) UV-visible absorbance spectra and (**c**) fluorescence emission spectra (λ_ex_ = 360 nm) of free Ce6 and DC in DMSO or DW (10 μg/mL Ce6).

To determine the improved hydrophilicity of DC, the solubility in DMSO and DW was assayed. Ce6 had good solubility in DMSO, but was not soluble in aqueous media, as shown in Figure 3a. However, DC exhibited significantly enhanced solubility in DW. In addition, the solubility of DC was confirmed based on UV-visible absorption. In DW, DC revealed a strong Soret band at 404 nm with four relatively weak Q bands at 508, 537, 612 and 665 nm because of its enhanced solubility in aqueous media (Figure 3b). These characteristic absorption bands are very similar to those of free Ce6 in DMSO, indicating that the Ce6 chromophore does not change upon conjugation. In addition, the fluorescence emission spectra of DC in DW and Ce6 in DMSO were similar, with maximum emission at 670 nm. However, the fluorescence intensity of DC in DW was lower than that of Ce6 in DMSO (Figure 3c). These results suggest that DC is a candidate for the clinical treatment of PDT.

### 3.2. Detection of Singlet Oxygen Production

**Figure 4:**
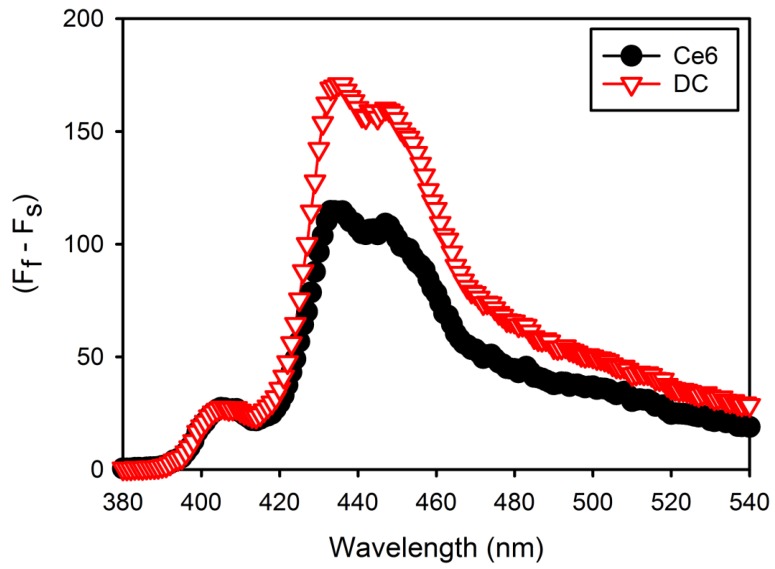
9,10-Dimethylnathracene (DMA) fluorescence change (F_f_ − F_s_) due to singlet oxygen generation by free Ce6 and DC after laser irradiation in Dulbecco’s phosphate-buffered saline (DPBS) (pH 7.4), where F_f_ and F_s_ represent the fluorescence intensity of full DMA and each sample.

^1^O_2_ generation from free Ce6 or DC in DPBS (pH 7.4) during laser irradiation was determined using DMA as the ^1^O_2_ trap. Fluorescent DMA reacts selectively with ^1^O_2_ to form the endoperoxide, causing the reduction in the fluorescence of DMA [16]. The changes in DMA fluorescence intensity (F_f_ − F_s_) were monitored in order to confirm ^1^O_2_ generation from free Ce6 or DC, resulting in DC generating higher ^1^O_2_ than free Ce6 in DPBS (Figure 4). This means that DC molecules are stabilized in DPBS, but free Ce6 molecules are partially aggregated in DPBS because of low water solubility.

### 3.3. Intracellular Uptake and Distribution of DC Nanoconjugate

**Figure 5:**
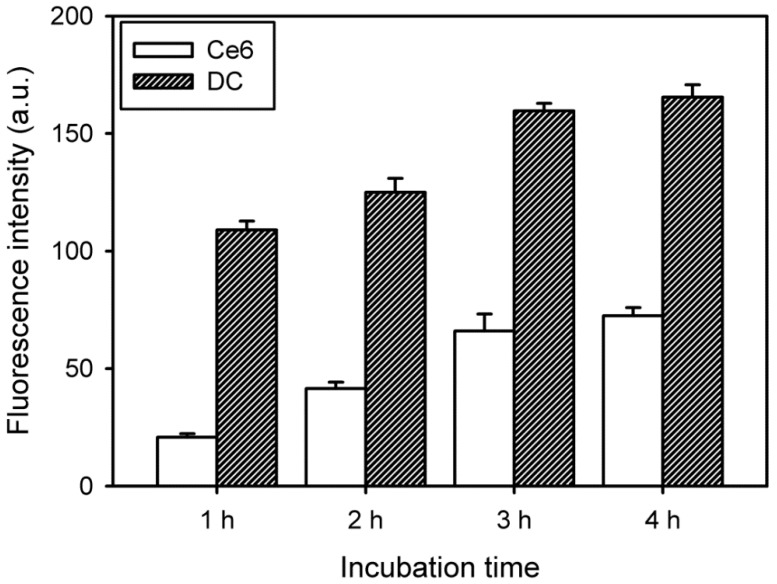
Change in fluorescence intensity due to intracellular uptake of free Ce6 and DC (4 μg/mL Ce6) in HeLa cells as a function of incubation time (*n* = 5).

Compared with conventional techniques, PDT is a less invasive form of cancer therapy. To investigate cytotoxicity, the cellular internalization of free Ce6 and DC into cancer cells was first assessed by using fluorescence spectroscopy combined with a multimode microplate reader. The samples contained HeLa cells incubated for 1, 2, 3 and 4 h. The initial intracellular uptake of DC was fast (within 3 h), with a subsequent slow increase, as shown in Figure 5. Furthermore, the intracellular uptake of DC was significantly higher than that of free Ce6.

**Figure 6:**
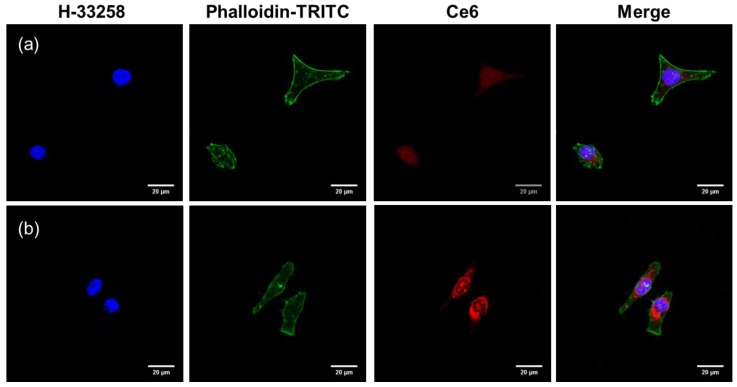
Confocal laser scanning microscopy image of the intracellular distribution of (**a**) free Ce6 and (**b**) DC in HeLa cells after incubation for 2 h in the dark.

Confocal microscopy provided more precise information on the intracellular distribution of free Ce6 and DC after cellular uptake. At 2 h after incubation, free Ce6 presented a weak red fluorescence signal because of inefficient cellular internalization (Figure 6). Compared with free Ce6, Ce6 molecules in DC exhibited significantly increased red fluorescence intensity after 2 h of treatment. In addition, the red spots of Ce6 molecules in DC were widely spread in the cytoplasm. These results indicate that the conjugation of Ce6 with PAMAM dendrimer may improve the drug uptake efficiency because of enhanced cell adhesion and cellular uptake of nanoconjugates [18]. Moreover, the high intracellular uptake of DC may be closely related to the high phototoxicity in the cancer cells because the main target of PDT is the major cellular organelles in the cytoplasm.

### 3.4. In Vitro Phototoxicity of DC Nanoconjugate

**Figure 7:**
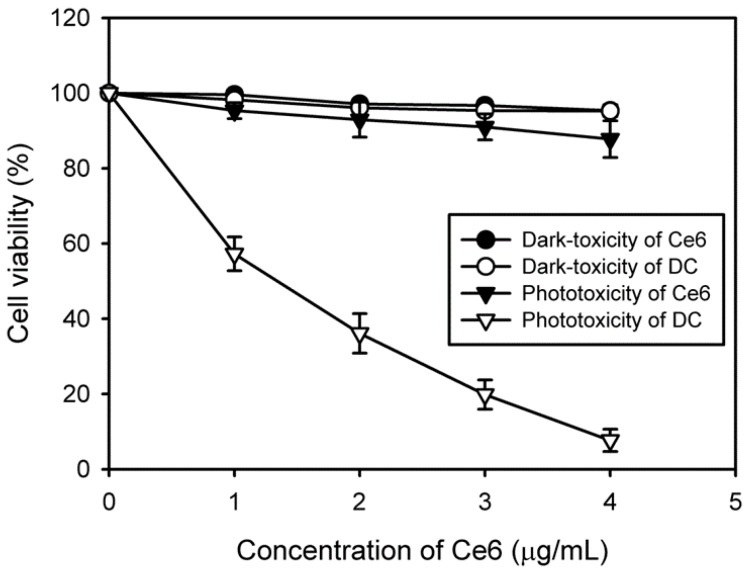
In vitro phototoxicity and dark-toxicity of various concentrations of Ce6 and DC on HeLa cells with and without laser irradiation (*n* = 6).

Upon irradiation with light of an appropriate wavelength, the photosensitizer is triggered to generate singlet oxygen for therapy; however, it should not exhibit cytotoxicity in the absence of light. To evaluate the in vitro phototoxicity of free Ce6 and DC, we assessed the viability of HeLa cells via the MTT assay after treatment with and without laser irradiation in the presence of free Ce6 and DC. The cell viability was normalized against the viability of control cells, which were not exposed to the drug and radiation. The results indicated no dark-toxicity in cells treated with free Ce6 or DC in comparison with the control cells, giving a cell viability over 95% (Figure 7). However, after laser irradiation, DC expressed higher phototoxicity compared with free Ce6, which might be due to an increased cellular uptake. As expected, the increased DC concentration caused more cell death. Approximately 92% of targeted cells were killed by 4 μg/mL of DC, clearly demonstrating the photodynamic activity of DC. These results suggest that, by increasing the cellular uptake of the drug, the conjugation of Ce6 with PAMAM dendrimer can improve the PDT efficacy of Ce6 in human cervical cancer cells.

**Figure 8:**
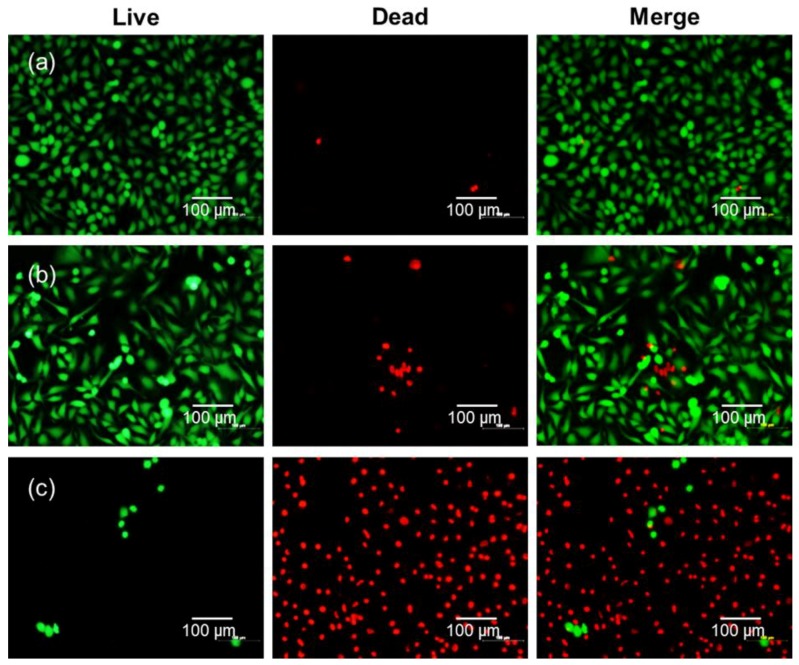
Fluorescence microscopy images of live HeLa cells and dead HeLa cells stained with calcein-AM (green) and ethidium homodimer-1 (EthD-1) (red) with (**a**) no drug, (**b**) free Ce6, and (**c**) DC (4 μg/mL Ce6) after irradiation by 2.5 J/cm^2^, 671 nm diode laser.

To confirm the improved PDT efficacy of DC, we further investigated cancer cell viability after treatment with free Ce6 or DC and laser irradiation by using a fluorescence-staining technique with calcein AM (green fluorescence) and EthD-1 (red fluorescence) to differentiate between live cells and dead cells. As shown in Figure 8, HeLa cells appeared green before laser irradiation in all samples, indicating live cells. However, HeLa cells treated with DC and subsequent laser irradiation appeared almost completely red due to cell death, although free Ce6 caused almost no cell death after laser irradiation. This is consistent with the result of the MTT assay.

**Figure 9:**
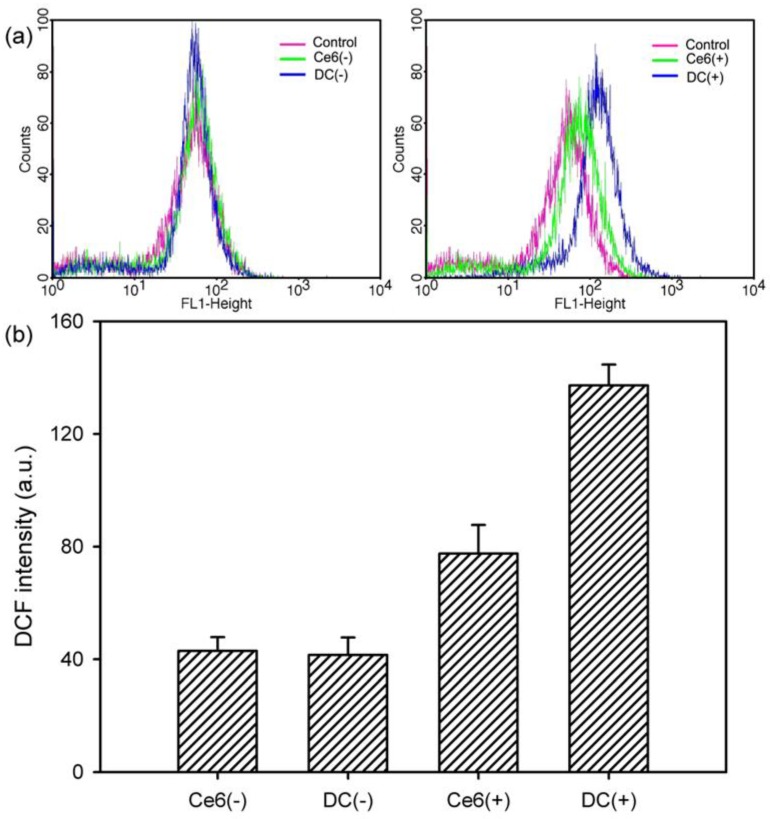
Flow cytometric analysis of ROS generation in HeLa cells treated with free Ce6 and DC (4 μg/mL Ce6). (**a**) Shift in fluorescence peak due to the ROS generation in the presence of free Ce6 and DC before irradiation (Ce6(−) and DC(−)) and after irradiation (Ce6(+) and DC(+)) with a 2.5-J/cm^2^, 671-nm diode laser. (**b**) DCF fluorescence intensity measured with the flow cytometer for investigating the ROS level with free Ce6 and DC before and after irradiation (*n* = 5).

The amount of ROS generated in HeLa cells was quantitatively and qualitatively assessed by applying fluorescence-activated cell sorting using a flow cytometer to support the PDT efficacy of DC. When exposed to light, the fluorescence peak did not shift due to the treatment with free Ce6 and DC (Figure 9a). Conversely, after laser irradiation, the fluorescence peaks shifted to the right because of the regeneration of the ROS. In addition, the peaks shifted further to the right upon treatment with DC, indicating that DC led to greater generation of ROS than did free Ce6. This result is also consistent with quantitative calculation, as shown in Figure 9b. When compared with non-irradiated samples (Ce6(−) and DC(−)), the DCF fluorescence intensity from the irradiated samples (Ce6(+) and DC(+)) increased. Among these samples, DC(+) revealed a significant increase in DCF fluorescence intensity, indicating elevated production of the ROS.

### 3.5. Induction of Cell Apoptosis by DC Nanoconjugate

**Figure 10:**
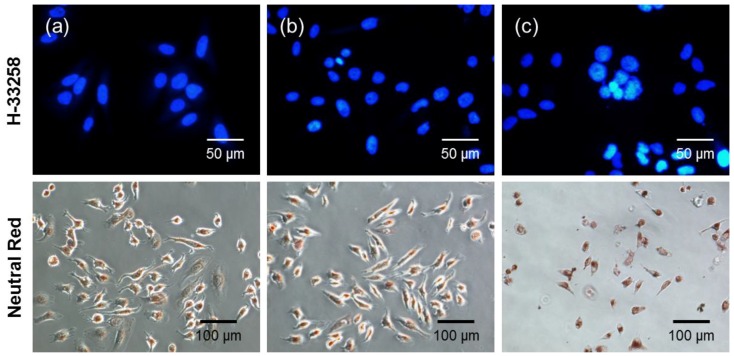
Morphology of HeLa cells stained with neutral red and H-33258 (**a**) before photodynamic treatment (no drug and no light) and after treatment with (**b**) free Ce6 and (**c**) DC (4 μg/mL Ce6), followed by irradiation with a 2.5-J/cm^2^, 671-nm diode laser.

PDT treatment can induce apoptosis, autophagy and necrosis pathways depending on the nature of the photosensitizers [1]. To further identify whether PDT with DC-induced cell death occurred via typical apoptosis or via necrosis of HeLa cells, the changes in overall and nuclear morphologies were visualized by using neutral red for the general morphology and H-33258 for chromatin DNA. As shown in Figure 10, a great number of cells were alive before the photodynamic treatment (i.e., no drug and no light). In addition, when cells were incubated with free Ce6 followed by laser irradiation, the nuclei and cytoplasm exhibited morphologies similar to that of the control cells, which in the case of most cells was a rounded morphology. However, after treatment with DC and laser irradiation, the rounded cells disappeared, and in their stead appeared volume-reduced cells with protuberances on the cell periphery. Finally, cells progressed through apoptosis, as determined by the observation of volume reduction, chromatin condensation and nuclear fragmentation, which are all typical apoptotic features.

## 4. Conclusions

Nanosized hydrophilic and biocompatible conjugates have received attention as a possible means of delivering photosensitizers. These nanoscale conjugates enhance the intracellular uptake of photosensitizers because of improved water solubility, leading to more efficient PDT. In this study, we propose a novel and simple reaction for preparing hydrophilic DC that uses a coupling agent in a one-step reaction between the amino and carboxyl groups. These nanoconjugates formed stable structures in aqueous solutions. The characteristic absorption bands of the prepared DC in DW were very similar to those of free Ce6 in DMSO, indicating that the Ce6 chromophore did not change upon conjugation. An increased intracellular uptake of DC into cancer cells induced higher phototoxicity of DC compared with free Ce6. In particular, DC-induced cell death occurred via typical apoptosis, which was confirmed by observing the changes in overall morphologies and nuclear morphologies. Based on these results, DC is a promising candidate to contribute to the development of a new generation of photosensitizer carriers for enhanced PDT treatment of cancers.

## Supplementary Materials

The following is available online at http://www.mdpi.com/2079-4991/8/6/445/s1: Figure S1: ^1^H NMR spectrum of DC.

## References

[1] Ruiz-González R., Acedo P., Sánchez-García D., Nonell S., Cañete M., Stockert J.C., Villanueva A. (2013). Efficient induction of apoptosis in HeLa cells by a novel cationic porphycene photosensitizer. Eur. J. Med. Chem..

[2] Cao W., Zeng X., Liu G., Li Z., Zeng X., Wang L., Huang L., Feng S.S., Mei L. (2015). Porphine functionalized nanoparticles of star-shaped poly(ε-caprolactone)-*b*-D-α-tocopheryl polyethylene glycol 1000 succinate biodegradable copolymer for chemophotodynamic therapy on cervical cancer. Acta Biomater..

[3] Hodgkinson N., Kruger C.A., Mokwena M., Abrahamse H. (2017). Cervical cancer cells (HeLa) response to photodynamic therapy using a zinc phthalocyanine photosensitizer. J. Photochem. Photobiol. B Biol..

[4] Deni E., Zamarrón A., Bonaccorsi P., Carreño M.C., Juarranz Á., Puntoriero F., Sciortino M.T., Ribagorda M., Barattucci A. (2016). Glucose-functionalized amino-OPEs as biocompatible photosensitizers in PDT. Eur. J. Med. Chem..

[5] Tao X., Yang Y.J., Liu S., Zheng Y.Z., Fu J., Chen J.F. (2013). Poly(amidoamine) dendrimer-graft porous hollow silica nanoparticles for enhanced intracellular photodynamic therapy. Acta Biomater..

[6] Yoon H.Y., Koo H.B., Choi K.Y., Lee S.J., Kim K.M., Kwon I.C., Leary J.F., Park K.N., Yuk S.H., Park J.H. (2012). Tumor-targeting hyaluronic acid nanoparticles for photodynamic imaging and therapy. Biomaterials.

[7] Park H., Na K. (2013). Conjugation of the photosensitizer Chlorin e6 to pluronic F127 for enhanced cellular internalization for photodynamic therapy. Biomaterials.

[8] Baek S.Y., Na K. (2013). A nano complex of hydrophilic phthalocyanine and polyethyleneimine for improved cellular internalization efficiency and phototoxicity. Colloid Surf. B.

[9] Ol’shevskaya V.A., Savchenko A.N., Zatsev A.V., Kononova E.G., Petrovskii P.V., Ramonova A.A., Tatarskiy V.V., Uvarov O.V., Moisenovich M.M., Kalinin V.N. (2009). Novel metal complexes of boronated chlorin e6 for photodynamic therapy. J. Organomet. Chem..

[10] Kesharwani P., Jain K., Jain N.K. (2014). Dendrimer as nanocarriers for drug delivery. Prog. Polym. Sci..

[11] De Freitas L.M., Soares C.P., Fontana C.R. (2014). Synergistic effect of photodynamic therapy and cisplatin: A novel approach for cervical cancer. J. Photochem. Photobiol. B Biol..

[12] Ahn T.G., Lee B.R., Kim J.K., Choi B.C., Han S.J. (2012). Successful full term pregnancy and delivery after concurrent chemo-photodynamic therapy (CCPDT) for the uterine cervical cancer staged 1B1 and 1B2: Preserving fertility in young women. Gynecol. Oncol. Rep..

[13] Choi M.C., Jung S.G., Park H., Lee S.Y., Lee C., Hwang Y.Y., Kim S.J. (2014). Fertility preservation by photodynamic therapy combined with conization in young patients with early stage cervical cancer: A pilot study. Photodiagnosis Photodyn. Ther..

[14] Hass R., Jacobs R., Kaufmann A.M., Hillemanns P., Soergel P. (2017). Sensitization of immune cells following hexylaminoevulinate photodynamic therapy of cervical intraepithelial neoplasia. Photodiagnosis Photodyn. Ther..

[15] Hu Q., Ding B., Yan X., Peng L., Duan J., Yang S., Cheng L., Chen D. (2017). Polyethylene glycol modified PAMAM dendrimer delivery of kartogenin to induce chondrogenic differentiation of mesenchymal stem cells. Nanomedicine.

[16] Kwag D.S., Oh N.M., Oh Y.T., Oh K.T., Youn Y.S., Lee E.S. (2012). Photodynamic therapy using glycol chitosan grafted fullerenes. Int. J. Pharm..

[17] Huang P., Lin J., Wang S., Zhou Z., Li Z., Wang Z., Zhang C., Yue X., Niu G., Yang M. (2013). Photosensitizer-conjugated silica-coated gold nanoclusters for fluorescence imaging-guided photodynamic therapy. Biomaterials.

[18] Li F., Na K. (2011). Self-assembled chlorin e6 conjugated chondroitin sulfate nanodrug for photodynamic therapy. Biomacromolecules.

