# Supplementary material for: Hydrophilic Chlorin e6-Poly(amidoamine) Dendrimer Nanoconjugates for Enhanced Photodynamic Therapy
Source: Nanomaterials (Basel). 2018 Jun 18;8(6):445. doi: 10.3390/nano8060445 (PMC6027052; doi:10.3390/nano8060445)
Supplement: Supplementary file 1 [file nanomaterials-08-00445-s001.pdf]

Supporting Information

Hydrophilic Chlorin e6-Poly(amidoamine) Dendrimer Nanoconjugates for Enhanced Photodynamic Therapy

So-Ri Lee and Young-Jin Kim*

Department of Biomedical Engineering, Daegu Catholic University, Gyeongsan 38430, Republic of Korea; esdkiss@naver.com (S.-R.L.); yjkim@cu.ac.kr (Y.-J.K.)

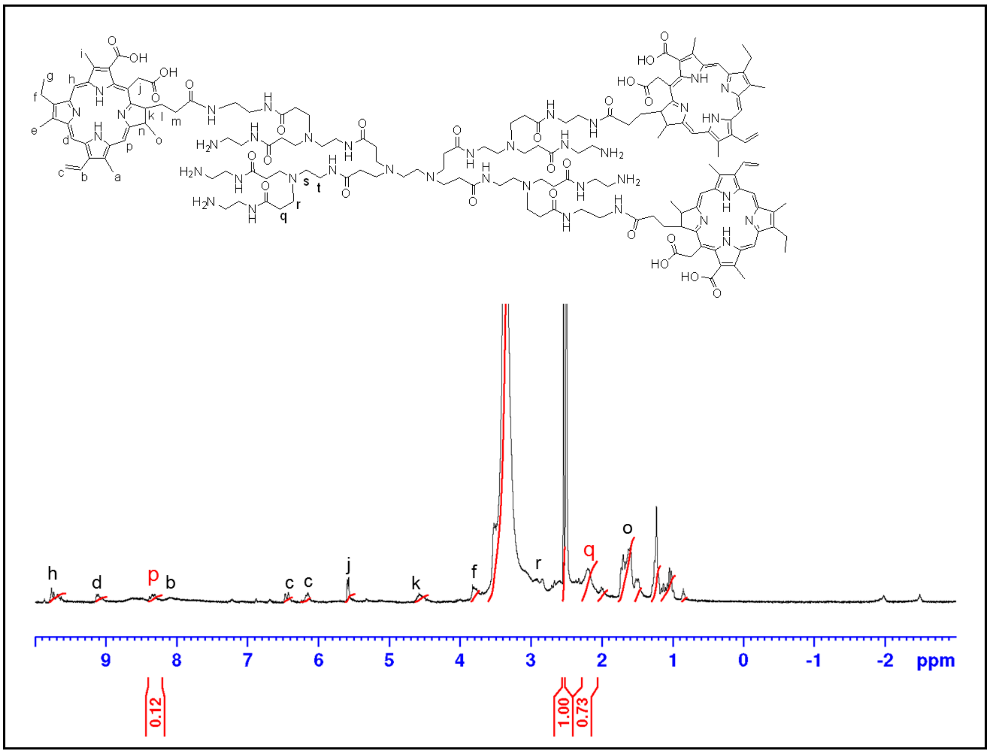

Figure S1. ^{1}H NMR spectrum of DC.
